# Anthropometric and Biochemical Profile of Children and Adolescents with Chronic Kidney Disease in a Predialysis Pediatric Interdisciplinary Program 

**DOI:** 10.1155/2015/810758

**Published:** 2015-01-13

**Authors:** Vanessa R. Silva, Cristina B. Soares, Juliana O. Magalhães, Isabella Peixoto de Barcelos, Debora C. Cerqueira, Ana Cristina Simões e Silva, Eduardo A. Oliveira

**Affiliations:** ^1^Nutrition Division, Hospital das Clínicas, Federal University of Minas Gerais (UFMG), 30130-100 Belo Horizonte, MG, Brazil; ^2^Pediatric Nephrourology Unit, Department of Pediatrics, UFMG, 31270-901 Belo Horizonte, MG, Brazil; ^3^Pediatric Branch, Interdisciplinary Laboratory of Medical Investigation, Faculty of Medicine, UFMG, Alfredo Balena Avenue 190, 2nd Floor, Room No. 281, 30130-100 Belo Horizonte, MG, Brazil

## Abstract

This is longitudinal retrospective observational cohort study that evaluated anthropometric and biochemical variables of children and adolescents admitted to a Predialysis Interdisciplinary Management Program (PDIMP) responsible for the follow-up of children and adolescents at stages 2 to 4 of chronic kidney disease (CKD) at a tertiary center. One hundred thirty-eight patients with CKD on predialysis treatment with median age at admission of 9 years and the median follow-up time of 5 years were evaluated. Seventy-four (53%) had CKD stage 3 at admission and 70 (51%) reached CKD stage 5 at the end of the follow-up. There was no significant difference between the mean initial and final hemoglobin and serum albumin. However, the final serum bicarbonate presented a significant improvement. Analyses stratified according to clinical variables of interest showed a significant improvement in body mass index (BMI) *Z* score, especially in the subgroup of children admitted under two years of age. In relation to stature-for-age *Z* score, data show a significant improvement in stature SD at the end of the study. In conclusion, the present study showed improvement of nutritional status of CKD patients and that the deterioration of renal function was not correlated with BMI-for-age *Z* score.

## 1. Introduction

International data on the incidence of pediatric patients at predialysis chronic kidney disease (CKD) range from 5.7 to 12 cases per million of age related population and the prevalence of 42.5 to 75 cases per million of age related population [1]. Pediatric patients constitute only a small proportion of the total population with CKD. However, the care of these patents represents a challenge for health care providers, which must pay attention not only to renal disease, but also to the various extrarenal manifestations that affect growth and development [2–5].

Continuous nutritional monitoring is a key component in the overall care of children and adolescents with CKD. Most infants with CKD will require some kind of nutritional intervention to achieve adequate growth. Common ways of nutritional approach start by oral salt and water supplements until gastrostomy and tube feeding [4, 6]. When planning nutritional intervention, the following five major components of the dietary prescription must be considered: energy, macronutrients, fluids and electrolytes, micronutrients, and calcium/phosphorus/vitamin D [7]. Nutritional care of these patients should be focused on maintaining adequate nutritional status, body mass index (BMI) *Z* score between −2 and +1, and height for age greater than or equal to −2 *Z* score [4, 8]. These goals should be achieved by providing a specific diet that also reduces the occurrence of uremic toxicity, metabolic abnormalities, and comorbidities related to CKD [4, 8, 9]. Conventional anthropometry remains to be the method most often used to monitor nutritional state in CKD patients [4, 8, 9].

In 1990, our Pediatric Nephrology Unit systematized a Predialysis Interdisciplinary Management Program (PDIMP) responsible for the follow-up of children and adolescents at stages 2 to 4 of CKD [10]. We have previously reported the clinical course of 107 children and adolescents admitted to our PDIMP and which were the independent predictors of progression to CKD stage 5 in our cohort [11–13]. In the present study, we extended our data by specifically focusing on the evaluation of anthropometric and biochemical variables during the follow-up of children and adolescents with CKD admitted to our PDIMP.

## 2. Patients and Methods

### 2.1. Study Design

This is a longitudinal retrospective observational cohort study that evaluated anthropometric and biochemical variables of children and adolescents at predialysis CKD from January 1990 to December 2008.

### 2.2. Patients

Children and adolescents of both genders, from birth to 19 years old, with glomerular filtration rate (GFR) equal to or below 75% of the value expected for age, according to reference values [10], and at least 6 months of follow-up at our PDIMP was included in this study. GFR was estimated by the formula of Schwartz et al. [14]. For the analysis of BMI, patients with serum albumin <3.5 g/dL and edema were excluded.

### 2.3. Ethical Aspects

The study was approved by the Ethics Committee of Federal University of Minas Gerais (UFMG), and the parents or persons responsible for the children gave written informed consent for them to participate.

### 2.4. Study Protocol

An interdisciplinary team, including pediatric nephrologists, pediatricians, nurses, psychologists, nutritionists, and social workers, was responsible for our PDIMP. After the initial investigation, patients were followed according to a systematic protocol described in detail in previous reports [12, 13]. The study protocol consisted of clinical evaluation, laboratory measurements, and anthropometric assessment of patients at admission and at the last visit in our PDIMP until the end of the analysis period. The visits were scheduled periodically at intervals of approximately 1 to 3 months, and a complete examination including measurements of blood pressure, height, and weight was performed on each occasion. Laboratory evaluation was also performed at approximately 1- to 3-month intervals, depending on the clinical condition of each patient. The routine exams were venous gas analysis, total peripheral blood count and serum levels of parathyroid hormone (PTH), urea, creatinine, sodium, potassium, chloride, calcium, phosphorus, and albumin. Conditions associated with CKD, such as anemia, hypertension, acidosis, renal osteodystrophy, and malnutrition, were carefully managed according to standard recommendations for pediatric patients at stages 2 to 4 of CKD [8, 15–18]. The prescribed diet provided at least 100% of the estimated average requirement (EAR) for energy for chronological age and at least 100% of the reference nutrient intake (RNI) for protein for height for age, based on the dietary reference values for food, energy, and nutrients for a general population [4, 19, 20]. Supplements of sodium bicarbonate, calcium carbonate, and calcium were given according to blood biochemistry findings. A vitamin D analog was used according to the values of serum PTH and following the guidelines of KDOQI [8, 15–18]. Therapeutic supplementation of oral iron was given for patients with saturation index of transferrin lower than 20% and ferritin lower than 100. Erythropoietin was available during the follow-up of the patients after 1990 and prescribed according to guidelines [18]. Food supplement was not used in our patients. Unfortunately, human recombinant growth hormone (hRGH) was not prescribed for our patients, since this treatment is still not available for CKD patients in Public Brazilian Healthy System. Antihypertensive drugs were routinely used for patients with blood pressure above the 95th percentile following standard recommendations [17, 21].

### 2.5. Baseline and Follow-Up Data

The data reviewed were obtained at patients' admission and at last visit. Clinical variables included primary renal disease, gender, race, age at admission, and blood pressure measurements. Laboratory variables were hemoglobin levels, PTH, serum albumin, phosphate, calcium, and bicarbonate. Anthropometric variables were weight, height, height for age *Z* score (HAZ), BMI, and BMI-for-age *Z* score. Weight was measured to the nearest 0.1 kg on calibrated digital scales. Standing height was considered as the average of three measurements, to the nearest millimeter, using a fixed stadiometer with the head aligned in the Frankfort horizontal plane. An infantometer was used to measure the height of patients with less than 2 years and/or with less than 1 meter. The software Anthro version 3.2.2 calculated *Z* score of BMI for age and height for age for children from 0 to 5 years, while the software Anthro Plus version 1.0.4 was used for children and adolescents above 5 years old. The classification of nutritional status was based on the cutoff points recommended by the World Health Organization as follows: BMI *Z* score < −3.00 severe thinness, > −3.00 and < −2.00 thinness, > −2.00 and < +1.00 normal, > +1.00 and < +2.00 overweight and > +2.00 and < +3.00 obesity, and > +3.00 severe obesity [22].

### 2.6. Statistical Analysis

A database in the statistical package SPSS version 17.0 was developed. Nutritional parameters were expressed as mean and standard deviation (SD). Normality of distribution was evaluated by Kolmogorov-Smirnov test for each parameter. The paired *t*-test was used to compare means of nutritional and biochemical parameters at baseline and at last follow-up visit. ANOVA was used for mean comparisons. Spearman correlation coefficient was used to assess the correlation between the deterioration of renal function and anthropometric variables. Values of *P* < 0.05 were considered statistically significant.

## 3. Results

A total of 138 children and adolescents with CKD on predialysis treatment were included in the analysis. The median age at admission was 9 years (interquartile range (IR), 2.3–13.2 years) and the median follow-up time was 58 months (IR, 23.3–94.7 months). Concerning primary renal disease, the majority of patients presented congenital anomalies of the kidney and urinary tract (*n* = 81, 58%), followed by glomerular diseases (*n* = 30, 21.7%) and cystic diseases (*n* = 20, 14.5%) and 8 (5.8%) patients had less common renal disorders. At baseline, hypertension was present in 57 (41%) patients and 73 (52%) children had proteinuria.

### 3.1. Clinical and Laboratory Features

Regarding renal function at admission, 74 (53%) of patients had moderate decrease in GFR (stage 3), 52 (37%) presented severe reduction (stage 4), and only 13 (10%) had mild reduction of GFR (classified as CKD stage 2). At baseline, the mean GFR was of 38.2 ± 15.6 mL/min/1.73 m^2^, whereas at last follow-up visit the mean GFR decreased to 28.3 ± 21.8 mL/min/1.73 m^2^.  Table 1 shows the main clinical and laboratory characteristics at baseline and at last follow-up visit. In spite of decline of GFR, biochemical and hematological parameters remained stable or even improved at last visit. For instance, albumin and hemoglobin values did not differ significantly as compared at baseline and at last visit. On the other hand, serum bicarbonate levels improved significantly at last visit, probably as a consequence of appropriate oral supplementation (Table 1).

### 3.2. Growth and Nutritional Features

**Figure 1:**
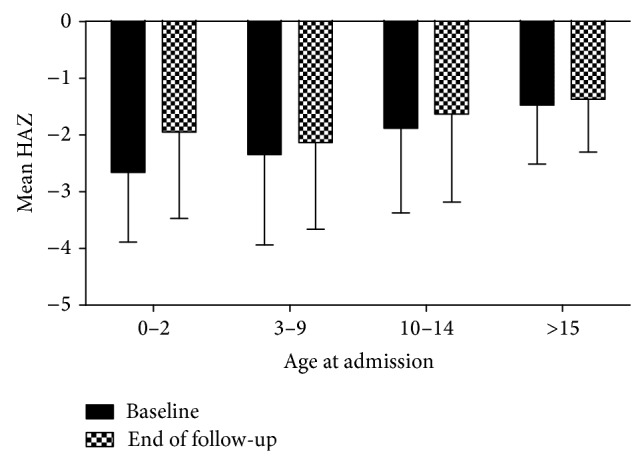
Mean values of height for age *Z* score (HAZ) at admission (baseline) and at last follow-up visit (end of follow-up) according to age at admission (expressed in years) for pediatric patients with chronic kidney disease.

At admission, HAZ was of −2.15 ± 1.46, whereas at last follow-up visit HAZ was of −1.82 ± 1.49, showing a significant improvement of final height (*P* = 0.001). Of note, at entry in the PDIMP, 40 patients (29%) presented a HAZ under −3.00, indicating extreme short stature. At last visit, the percentage of patients with extreme short stature decreased to 18% (25 patients). At admission, 75 (54%) patients had HAZ under −1.88, equivalent to less than the third percentile, while at last follow-up visit this percentage of patients reduced to 46.4% (64 children). Stratified analyses according to clinical variables of interest showed that some subgroups presented significantly greater deficit of stature. Regarding age of admission, HAZ was significantly lower for infants as compared to patients enrolled after this age (−2.67 ± 1.21 versus −2.00 ± 1.49, *P* = 0.02). Considering age at admission stratified into four groups (0–2 years, 3–9 years, 10–14 years, and >15 years), significant differences were found in mean HAZ among groups. Significant differences were detected in the comparisons between infants and patients of 10 to 14 years (*P* = 0.016) and between infants and patients above 15 years (*P* = 0.007). In addition, mean HAZ also differed in the comparison between children aged 3 to 9 years to patients above 15 years of age (*P* = 0.037). On the other hand, it should be noticed that at last follow-up visit, no significant differences were detected among all age groups (*P* = 0.18).  Figure 1 illustrates the improvement of HAZ at last follow-up visit for all age groups, mostly for infants.

At entry, among 119 patients evaluated and classified according to BMI *Z* score for age, 74.8% (*n* = 89) were considered as normal, 5.9% (*n* = 7) had severe thinness, 12.6% (*n* = 15) had thinness, 4.2% (*n* = 5) were overweight, and 2.5% (*n* = 3) exhibited obesity. At last follow-up visit, 79.8% (*n* = 95) of patients were normal, 5% (*n* = 6) had severe thinness, 10.1% (*n* = 12) had thinness, 3.4% (*n* = 4) were classified as overweight, and 1.7% (*n* = 2) patients presented obesity. The initial and final BMI *Z* score did not significantly differ. The means of BMI *Z* score were −0.866 ± 1.29 at entry and −0.860 ± 1.21 at last visit (*P* = 0.96).

**Figure 2:**
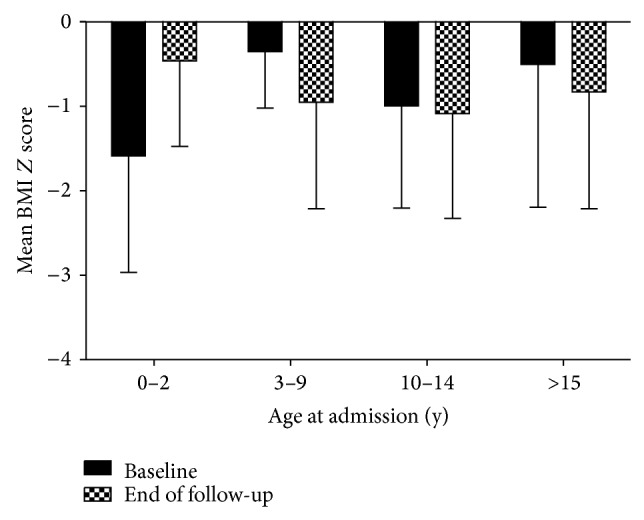
Mean values of body mass index (BMI) *Z* score at admission (baseline) and at last follow-up visit (end of follow-up) according to age at admission (expressed in years) for pediatric patients with chronic kidney disease.

However, stratified analyses according to clinical variables of interest showed that some subgroups exhibited significant improvement in nutritional status. Regarding the age of admission, there was a significant difference between the initial and final BMI *Z* score.  Figure 2 shows an improvement in BMI *Z* score in the subgroup of children admitted under two years of age. In this subgroup, the mean BMI *Z* score at admission was −1.58 ± 1.37, while, at last visit, BMI *Z* score was −0.46 ± 1.01 (*P* = 0.001). On the other hand, in the subgroup of children admitted between 3 to 9 years old, there was a significant decrease in mean BMI *Z* score at admission when compared to BMI *Z* score at last follow-up visit (−0.25 ± 0.76 versus −0.95 ± 1.26, *P* = 0.001). However, it should be pointed that both indexes were within the normal range.

**Figure 3:**
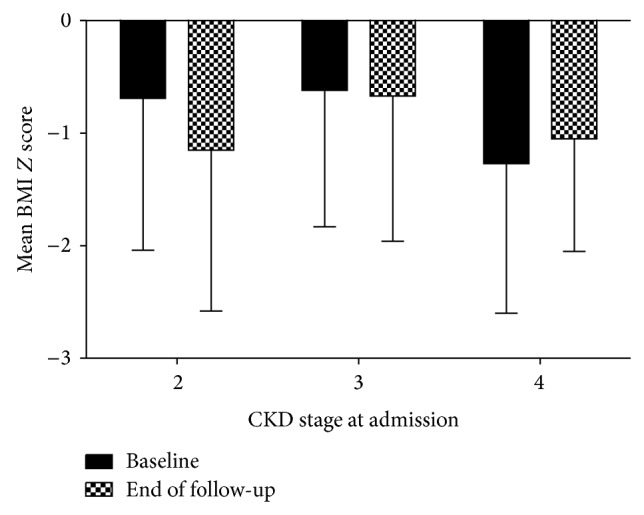
Mean values of body mass index (BMI) *Z* score at admission (baseline) and at last follow-up visit (end of follow-up) according to chronic kidney disease (CKD) stage at admission.

Considering CKD stages at baseline, BMI *Z* score significantly differed according to CKD stage (*P* = 0.032). At admission, BMI *Z* scores for CKD patients at stages 2, 3, and 4 were, respectively, −0.69 ± 1.35, −0.62 ± 1.21, and −1.27 ± 1.33. However, these differences were not observed at last follow-up visit (CKD stage 2 = −1.15 ± 1.43, CKD stage 3 = −0.67 ± 1.29, and CKD stage 4 = −1, 0.5 ± 1.0, *P* = 0.203 for multiple comparisons).  Figure 3 shows a slight worsening in BMI *Z* score for the subgroup of patients enrolled at CKD stage 2 and a small improvement in BMI *Z* score, especially for patients admitted at CKD stage 4, although these differences did not reach statistical significance (*P* = 0.333).

**Figure 4:**
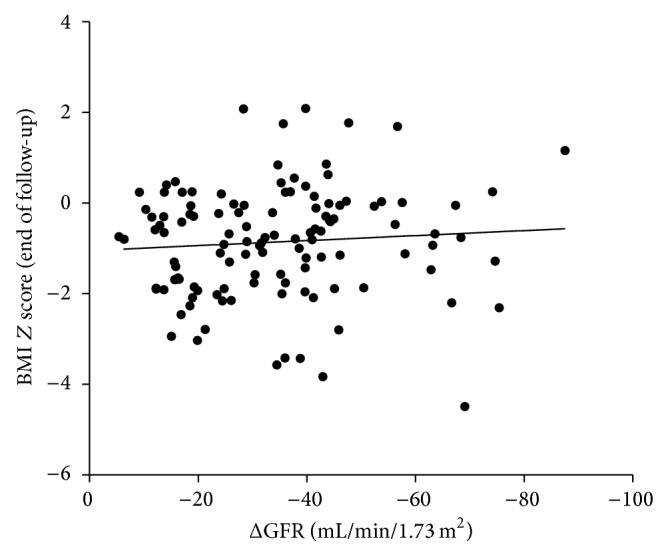
The absence of correlation between the decline of glomerular filtration rate (ΔGFR) and the body mass index (BMI) *Z* score at last follow-up visit (end of follow-up) (*r* = −0.078, *P* = 0.40).

Of 138 patients, 70 (51%) reached CKD stage 5 at last follow-up visit, whereas 52 37.7% remained in predialysis treatment, 7 (5.1%) were transferred to other services, 6 (4.3%) abandoned treatment, and 3 (2%) patients died. In spite of the expected decline of renal function for the majority of patients, deterioration of renal function was not significantly correlated with nutritional status at last follow-up visit (*r* = −0.078, *P* = 0.40) (Figure 4).

## 4. Discussion

In this retrospective cohort study of children and adolescents with CKD admitted to a predialysis pediatric interdisciplinary program we reported anthropometric and biochemical data at baseline and at last follow-up visit. During the follow-up of these patients, despite the decline of renal function, biochemical data significantly improved, especially bicarbonate and serum phosphate. Although at last visit about half of patients reached CKD stage 5, nutritional status remained at least stable. Furthermore, growth parameters and in BMI *Z* score significantly improved in the subgroup of children admitted under two years old.

The etiology of CKD in our study was similar to that reported in other series, in which there was a predominance of CAKUT [23, 24]. While the dominant causes of CKD in adults are diabetic nephropathy and hypertension, nearly 60–70% of children affected with CKD have congenital or inherited kidney disorders [1, 5]. In our series, CAKUT and inherited kidney disorders account for about 70% of CKD etiology. Irrespective of primary cause of renal damage, the onset of CKD triggers a chain of events with a common final pathway where preterminal kidney damage progresses to kidney failure [2, 25–28]. Accordindly, about half of our patients reached CKD stage 5 in a median of 5 years of follow-up.

In pediatric CKD, growth retardation is a frequent and important problem [4, 20]. Patients undergoing predialysis or dialysis treatment, or even after transplantation, have reduced growth rate, which tends to be more intense at final stages of renal disease. The North American Pediatric Renal Trials and Collaborative Studies (NAPRTCS) reports that at the time of entry to the registry, 36.6%, 47.0%, and 43.0% of children with predialysis CKD, dialysis, and transplantation, respectively, have severe short stature, arbitrarily defined by the authors as a standard deviation score (SDS) of less than −1.88 [29–31]. In our series, by these criteria, more than half of our patients had severe short stature at entry and 46.4% remanied with this condition at the final assessment. The etiology of growth delay in children with CKD is clearly multifactorial. Several factors have been proposed by previous studies as risk factors; these include age at onset of CKD, primary renal disease, protein calorie malnutrition, increased protein catabolism, metabolic acidemia, renal osteodystrophy, anemia, urinary sodium losses, and other electrolyte abnormalities [19, 30, 32, 33]. Interestingly, in our study, children under 2 years old had significant lower HAZ at admission to our PDIMP. However, a remarkable improvement in growth rate was observed for this subgroup of children at last follow-up visit. Consequently, at the end of the follow-up, no differences in HAZ *Z* score were detected between all subgroups of age. The improvement observed in patients admitted under two years old may be related to early correction of factors that contribute to growth retardation. Unfortunately, in spite of strict management of CKD by our PDIMP, almost half of our patients had severe short stature at the last assessment. Some infants and children with CKD did not develop properly despite adequate nutrition and appropriate metabolic control. These patients will probably benefit from the prescription of recombinant growth hormone (rhGH). In this regard, the 2006 US Consensus Statement on Assessment and Treatment of Short Statue in Pediatric Patients with CKD recommends rhGH therapy for CKD children when all other potential causes of growth failure have been properly evaluated and corrected. Indeed, the requirement of daily injections and psychosocial factors have been associated with low use of rhGH therapy [29, 31, 34]. It should be pointed that the majority of our CKD patients who were clearly eligible for rGH therapy did not receive it. The main reasons for this fact were the high costs of rhGH therapy and the unavailability of rhGH for pediatric CKD patients at the Public Health System (SUS) in Brazil. In this context, Al-Uzri et al. [35] have recently reported a significant positive association between both catch-up growth and rhGH use on parent-proxy reports of physical and social functioning in children with CKD. Thus, the improvement of quality of life reinforces the importance of interventions to recover height in children with CKD.

Adequate nutrition is a concern for all children with CKD in part due to the impact of nutritional intake on growth and neurodevelopment [4, 20, 36]. Previous studies in adults with CKD stage 5 have demonstrated that poor nutritional status is a risk factor for infectious complications and death [37, 38]. Nutritional status is also an important prognostic factor in pediatric CKD patients. Malnourished patients are at risk of increased morbidity and mortality and worse quality of life compared with their normal-weight counterparts [39–42].

Wong et al. [41] have evaluated the association between anthropometric measurements and death among pediatric patients at CKD stage 5 by using data of 1,949 patients from the Pediatric Growth and Development Special Study (PGDSS). These authors showed that each decrease by 1 SDS in height was associated with a 14% increase in the risk of death and for each 1 SDS decrease in growth velocity among patients in their sample, the risk of death increased by 12%. Our analysis showed that the majority of patients had normal BMI at entry according to WHO classification and most children and adolescents remained with good nutritional status at last follow-up visit in spite of the inevitable decline of renal function.

Of great interest, our study showed that the subgroup of infants significantly improved BMI *Z* score at last follow-up visit in comparison to values at admission. As recently pointed out by Foster et al. [7], attention to nutritional intake is of particular importance in infants and very young children with CKD. Growth and neurodevelopment progress most rapidly during the first 3 years of life; therefore, inadequate nutrition during this critical period may result in serious growth restriction and developmental delays [7]. Nutritional care of this vulnerable population requires the collaborative efforts of a multidisciplinary team including nephrologists, renal nurses, and a trained dietician. The importance of nutritional care is clearly supported by a recent study of Slinin et al. [43] with adult and elderly patients who started hemodialysis therapy between June 2005 and May 2007 in the United States. These authors found an independent association between nutritional care for more than 12 months prior to the start of dialysis and lower mortality during the first year of dialysis.

Our results must be considered in light of potential limitations associated with the retrospective design of our study. For instance, a factor that is often overlooked in the assessment of growth in children with CKD is genetic potential, determined by average parental height [44]. Unfortunately, this factor cannot be assessed in our analysis once these data were not available in the patients' records. Another limitation is the use of BMI as a marker of nutritional status in this population. It should be considered that BMI does not allow the distinction between fat mass and free-fat mass and an appropriate BMI for age does not necessarily indicate optimal body composition. Weight gain may be due to excess fat, consequently with an imbalance between fat and lean mass. For instance, in a cross-sectional study using dual-energy X-ray absorptiometry, Gao et al. [45] concluded that expressing BMI relative to chronologic age results in significant underestimation of relative lean mass and adiposity in children and adolescents with CKD and may result in overdiagnosis of underweight.

Nevertheless, in spite of judicious management of nutritional factors, anemia, serum phosphorous/calcium balance, and metabolic acidosis, approximately 46% of our patients had severe to moderate growth failure (HAZ < −2.00) at last follow-up visit. The low socioeconomic status of our population, the older age at admission, and the unavailability of rhGH in Brazilian Public Healthy System may negatively impact on the growth of our patients. In conclusion, despite the decline of renal function, our study showed that an appropriate nutritional care may contribute to appropriate growth and recovery of nutritional status in pediatric CKD patients, particularly for infants.

## Figures and Tables

**Table 1:** Clinical and laboratory data at admission and at last follow-up visit.

Variables	At admission	At last follow-up visit	*P* values
GFR^*^ (mL/min/1.73 m^2^)	38.2 ± 15.6	28.3 ± 21.8	<0.001
Albumin (g/dL)	3.97 ± 0.80	4.00 ± 0.73	0.552
Hemoglobin (g/dL)	11.10 ± 2.13	11.04 ± 2.36	0.783
Potassium (mEq/L)	4.76 ± 0.73	4.82 ± 0.80	0.460
Calcium (mg/dL)	9.37 ± 1.03	9.29 ± 0.95	0.422
Phosphorus (mg/dL)	5.22 ± 1.20	4.99 ± 1.24	0.089
Serum bicarbonate (mEq/L)	19.59 ± 4.77	22.00 ± 4.42	<0.001

^*^GFR: glomerular filtration rate estimated by the formula of Schwartz et al. [14].
